# C-reactive Protein in Patients with Metastatic Clear Cell Renal Carcinoma: An Important Biomarker for Tumor-associated Inflammation

**Published:** 2007-02-07

**Authors:** Albrecht Reichle, Jochen Grassinger, Klaus Bross, Jochen Wilke, Thomas Suedhoff, Bernhard Walter, Wolf-Ferdinand Wieland, Anna Berand, Reinhard Andreesen

**Affiliations:** Department of Hematology and Oncology, University Hospital of Regensburg.Department of Urology, University Hospital of Regensburg, Germany

**Keywords:** Anti-inflammatory therapy, Interferon-alpha, PPARgamma, COX-2

## Abstract

Two consecutive multi-center phase II trials were designed to prove the hypothesis, whether therapeutic modeling of tumor-associated inflammatory processes could result in improved tumor response.

Therapy in both trials consisted of low-dose capecitabine 1g/m2 twice daily p.o. for 14 days, every 3 weeks, day 1+, and rofecoxib 25 mg daily p.o., day 1+ (from 11/04 etoricoxib 60 mg daily instead) plus pioglitazone 60 mg daily p.o., day 1+. In study II low-dose IFN-α 4.5 MU sc. three times a week, week 1+, was added until disease progression.

Eighteen, and 33 patients, respectively, with clear cell renal carcinoma and progressive disease were enrolled. Objective response (48%) was exclusively observed in study II (PR 35%, CR 13%), and paralleled by a strong CRP response after 4 weeks on treatment, p = 0.0005, in all 29 pts (100%) with elevated CRP levels. Median progression-free survival could be more than doubled from a median of 4.7 months (95% CI, 1.0 to 10.4) to 11.5 months (6.8 to 16.2) in study II, p = 0.00001. Median overall survival of population II was 26 months.

Efficacious negative regulation of tumor-associated inflammation by transcription modulators may result in a steep increase of tumor response and survival.

## Introduction

Increased C-reactive protein (CRP) levels in serum may be frequently observed in patients with metastatic clear cell renal carcinoma (CCRC) [Ljungberg et al. 1997]. CRP as acute phase protein indicates the hepatocytes’ response to a systemic inflammatory activity mediated by pro-inflammatory cytokines (IL-6, IL-1, TNF-α) [Yoshida et al. 2002]. Particularly in CCRC, CRP may be additionally derived from the tumor cells itself [Jabs et al. 2005]. The presence of acute phase response as a negative predictor for survival is obvious [Bromwich et al. 2004; Kerr, 2006; Thiounn et al. 1997].

Hypoxia is an omnipresent phenomenon in growing tumors [Yan et al. 1995]. Particularly in CCRC the ‘hypoxic phenotype’ is based on mutations or gene silencing of the ‘Von-Hippel-Lindau’ tumor-suppressor gene [Herman et al. 1994; Latif et al. 1993]. Genes induced by the consecutive accumulation of the transcription factor hypoxia-inducible factor alpha (HIF-α) include the vascular endothelial growth factor (VEGF), the platelet-derived growth factor, and NF-kappaB among others, providing targets for an combined angiostatic and anti-inflammatory therapy in CCRC [Cummins and Taylor, 2005; An and Rettig, 2005]. Therefore, CCRC possesses model character for pharmacological interventions aimed to attenuate inflammation and neoangiogenesis, e.g., with COX-2 inhibitors, glitazones, and IFN-α [Panigrahy et al. 2005; Prasad, 2006; Tilg et al. 1995].

Therapy of metastatic renal cell carcinoma (RCC) has to face a vexing problem: The resistance against currently available therapeutic approaches. Continuous production and release of pro-inflammatory cytokines in metastatic CCRC, particularly IL-6, by tumor and adjacent stroma cells may account in part for the drug resistance [Galban et al. 2003; Angelo et al. 2002; Hodge et al. 2005]. Little is known of how pro-inflammatory cytokines such as IL-6 serve to promote growth of metastatic tumors. Serum IL-6 levels, however, are well correlated to metastatic stage and ECOG status [Mantovani et al. 2002].

Recently published data on the combined activity of pioglitazone and COX-2 inhibitors in metastatic cancer approved that cancer encompasses other diseases, such as inflammation: Similarly to non-malignant typically inflammation-linked diseases, pioglitazone (peroxisome proliferator-activated receptor-gamma, PPAR-γ agonist) may induce response in malignant diseases such as e.g., vascular sarcomas while attenuating the host’s inflammatory reaction to metastatic disease [Vogt et al. 2003; Reichle et al. 2004]. Moreover, results of a randomized phase II trial in metastatic melanoma are demonstrating that the survival rate may be significantly enhanced by the addition of anti-inflammatory therapy (pioglitazone plus COX-2 inhibitor) to low-dose continuous (metronomic) chemotherapy [Reichle et al. 2005].

The two consecutive phase II trials are based on our recent experiences on anti-inflammatory and angiostatic therapy in quite different metastatic malignancies [Hafner et al. 2005] and are aimed to study whether attenuation of tumor-related inflammation by pioglitazone plus/minus IFN-α may result in an improved response rate in CCRC [Pascual et al. 2005; Tilg et al. 1995]. The biomodulatory therapy in both trials is supplemented by low-dose chemotherapy (supposed angiostatic activity) with capecitabine [Oevermann et al. 2000].

## Patients and Methods

Participating centers in the trial were the Department of Hematology and Oncology and the Department of Urology of the University Hospital of Regensburg, Departments of Hematology and Oncology of the Hospitals Fürth and Passau.

### Eligibility

The local ethics committee approved the study protocol and patients were required to provide written informed consent before enrolment. Eligible patients were required to have progressive metastatic (according to RECIST requirements), locally recurrent or contra-lateral unresectable CCRC. If nephrectomy was not indicated due to missing operability clear cell histology was confirmed at a metastatic site. Patients with primarily metastatic disease experienced nephrectomy at least 21 days before the initiation of protocol treatment. In these patients disease progression was no prerequisite for the start of therapy. Brain metastases were no exclusion criteria, if those were controlled by surgery or radiotherapy prior to the start of study medication.

Patients were allowed to have received an unlimited number of previous systemic therapies including chemotherapy and/or immunotherapy or antiangiogenic agents such as thalidomide and IFN-α (IFN-α pretreatment was no exclusion criterion, because we suggested a synergistic anti-inflammatory activity of pioglitazone/COX-2 inhibitor/IFN-α). Previous treatment with pioglitazone or capecitabine was an exclusion criterion. The remaining inclusion criteria included those of the Eastern Cooperation Oncology Group (ECOG) (with the exception of serum creatinine ≤ 1.5 mg/dL).

### Pre-treatment evaluation

Aside from the acquisition of a medical history, baseline evaluation included a physical examination, an assessment of ECOG performance status, a complete blood cell count, serum chemistry assays, coagulation tests, a chest X-ray, abdominal ultrasound scanning, computed tomography (CT) (scanning of the thorax and abdomen, and facultative bone scanning or CT scanning of the brain if metastasis were clinically suspected).

Patients subsequently were monitored before the start of chemotherapy and every 3 weeks thereafter (assessment of toxicity, serum chemistry assays, one of which measured CRP levels, and a physical examination). For patients continuing study medication target lesions were assessed (via abdominal ultrasound or chest X-ray) before each 3-week therapy cycle. If these techniques suggested response to treatment or progressive disease, CT scans were performed before the routinely scheduled response evaluations with CT scans in 12-week intervals.

### Treatment

Patients in study I received 1 g/m^2^ oral capecitabine (Roche) administered twice daily from day 1+, 60 mg oral pioglitazone (Takeda) and 25 mg oral rofecoxib (MSD) daily starting with day 1+. From November 2004 we substituted rofecoxib for 60 mg oral etoricoxib (MSD) daily (rofecoxib was withdrawn from the market). Patients in study II received medication of study I plus 4.5 MU IFN-α sc. (Roche) 3 times per week, from day 1+.

Treatment was continued until disease progression was documented or for a maximum of 6 weeks after confirmation of complete remission.

### Efficacy assessment

Response was evaluated in patients who had a follow-up duration of ≥ 3 weeks by the treating physicians, and centrally (blinded) by the imaging unit of the university hospital of Regensburg. Response categories were assigned by using the **R**esponse **E**valuation **C**riteria in **S**olid **T**umors (RECIST) criteria [Gehan and Tefft, 2000]. All major responses were reconfirmed in 4- to 6-week intervals. Data reported represent the best response obtained during treatment according to study protocol.

### Dose modification

Drug administration was interrupted for respective grade 2 or 3 toxicity and resumed at a reduced dose on resolution to less than grade 2. In cases of reoccurrence of dose-limiting grade 3 or 4 toxicity the corresponding drug was discontinued.

Capecitabine therapy was continued with 75% starting dose for the first, and 50% for the second occurrence. IFN-α administration was continued at a dose of 3 MU three times a week; COX-2 inhibitor administration at a dose of 12.5 mg rofecoxib or 60 mg etoricoxib every second day; and pioglitazone at a reduced dose of 45 mg.

According to experiences in previous phase II studies dose modification of pioglitazone was not performed as long as a dose reduction or discontinuation of the COX-2 inhibitor was sufficient to resolve edema and/or renal insufficiency to < grade 2.

### Statistical considerations

The current non-randomized phase II trials were designed to assess [1] response, [2] the qualitative and quantitative toxicity of the treatment schedules and [3] CRP response.

The case calculation for each study based upon available response data following treatment with single agents or combinations of drugs administered. We assumed a response rate ≥ 25% to proceed with the further development of the respective schedule [Creagan et al. 1991; Zaniboni et al. 1989; Haarstad et al. 1994]. Prerequisite to resume schedule II was a CRP decline > 30% in patients experiencing stable disease or objective response on schedule I independently of whether stage two of a two stage flexible design may be achieved with schedule I [Chen and Ng, 1998].

With the assumption of an undesired response rate of 5% the rejection boundary is then one response, if the sample size is 17 patients. If the observed responses are > 1, we have to enroll 28 patients at the second stage. Achievement of 7 responses among 28 patients indicate a true response rate > 25%. The level of this design is 5% and the power 0.9.

Progression-free survival was defined as the interval between the beginning of treatment and disease progression. Overall survival was calculated from the initiation of treatment until death or until November 2005 (date of final data analysis), which ever came first. Survival distribution was generated using Kaplan-Meier method. Survival analyses were performed on the intent-to treat population. Patients who withdrew from the study due to treatment related side effects were considered as treatment failure.

Comparison of survival for subsets of patients was accomplished by using two-sided log-rank analysis. In addition, the ‘Fischer’ exact and the ‘Student t’ – test were used to identify significant association between chemical and biologic variables.

## Results

### Patients characteristics

In total, 18 patients (of 2 centers) and 33 patients (of 4 centers) with unresectable CCRC were enrolled onto the study protocols between February 2002 and January 2003 (study I) and February 2003 and April 2005 (study II), respectively. Detailed patient characteristics are listed in Table 1. The age distribution in both cohorts corresponds to the age-related incidence of renal cell carcinoma, and a typical metastatic pattern was documented. Only a small proportion of patients did not undergo radical nephrectomy (11%/9%, study I/II). Twelve and 23 patients in cohort I/II (67%/70%) received either previous surgery of metastases (17%/52%), and/or systemic treatment (33%/19%), and/or radiotherapy (17%/18%).

Retrospectively the patients were categorized according to the clinically guided Motzer score and to the Leibovich score which is adapted to stage, metastatic site and histological qualities [Motzer, 2003; Leibovich et al. 2005]. The two cohorts differed neither in the Motzer nor in the Leibovich categories significantly (Table 1).

### Treatment

All patients in study I/II received at least three 3-week cycles of study medication. Two patients of protocol II (6%) were withdrawn from response analysis because these patients received up-front alternative treatments, one on of her own decision another met one of the exclusion criteria (coumarin therapy).

The median duration of treatment in study II was 11.5 months (95% CI, 7.3–15.7 months). At present, 16 patients (52%) are still on treatment for 8.5+ to 23.5+ months including 4 patients with stable disease for 8.5–21.2 months. An additional two patients with continuous CR (protocol II) are still without study medication for 4.1+ and 7.3+ months. In study I, the median duration of treatment was 4.7 months (95% CI, 0.9–10.4 months).

### Treatment efficacy

All 18 patients in protocol I (100%), and 31 of 33 patients in protocol II (94%) were assessable for response. No objective response occurred in protocol I, whereas 15 of the 31 patients assessable for response in protocol II (48%) had objective response (4 complete responses, 11 partial responses) including one pathologically confirmed complete response of a bone metastasis (fracture of a vertebra-body) (Table 2). Objective responses were diagnosed after a median time of 4.1 months (range 3.1 to 8.7 months). Responses occurred at all major tumor localizations (lung, pancreas, lymphnodes, liver, bone, contra-lateral kidney).

The metastases of patients with complete response were localized in the lung (n = 3), liver (n = 1), bone (n = 1), and in lymph nodes (n = 4). All these patients received prior tumor nephrectomy. At first relapse a chemoembolization of a metastasis was performed in one patient, another patient underwent surgical stabilization of a fracture of a vertebra-body and radiation of a further bone metastasis prior to study inclusion.

An additional 14 patients of protocol II had stable disease. Three of these patients had a measurable reduction of the metastatic lesions’ size but did not meet the RECIST response criteria. Best response in study I was stable disease in 9 patients (50%), another 9 patients suffered from continuous tumor progression. Only 2 of 31 patients (5%) had progressive disease on regimen II. All patients of protocol I/II (94%/24%) died of tumor progression.

**Figure 1:**
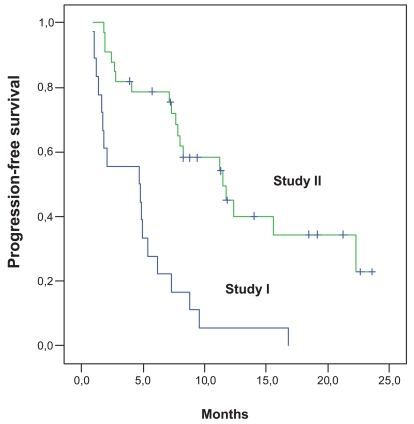
**Progression-free survival (Study I: N = 18; Study II: N = 33).** The median, 12 and 24 months progression-free survivals are 4.7 months, 13%, and 0% (study I) vs. 11.5 months, 49% and 24%, respectively (study II).

**Figure 2:**
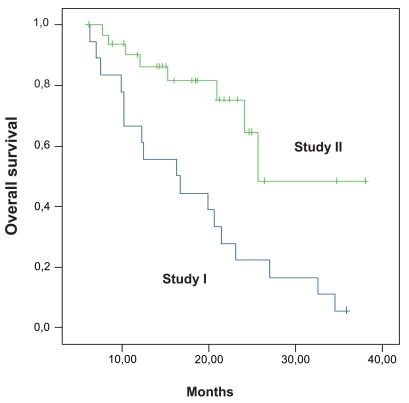
**Overall survival (Study I: N = 18; Study II: N = 33).** The median, 12, 24, and 36 months survivals are 16.2 months, 61%, 21% and 5% (study I) vs. 25.6 months, 88%, 65% and 48%, respectively (study II).

Progression-free survival of all patients enrolled on protocol I (n = 18), and protocol II (n = 33) is shown in Figure 1. After a median follow-up of 21 months/22 months (study I/II) the median, 12- and 24-month progression-free survivals were 4.7 months, 13%, and 0% vs. 11.5 months, 49%, and 24%. The overall survival is shown in Figure 2. The median, 12-, 24- and 36-month survivals were 16.2 months, 61%, 21%, and 5% vs. 25.6 months, 88%, 65%, and 48%, respectively.

The objective response rate for assessable patients of study II who did or did not receive previous systemic therapy (n = 6; n = 25) was 33% and 52%, respectively. Two responders received previously IFN-α. Progression-free survival rate was significantly improved only for untreated patients of cohort I (p = 0.03). The respective overall survival rates were not significantly different within both cohorts (p = 0.12).

Objective response to treatment was observed in all Motzer and Leibovich risk categories. Patients in low-, intermediate-, and high-risk categories (Motzer score) had major response of 60%, 47% and 33%, respectively, patients in Leibovich category −5 to −1, 0 to 2, 3 to 6, 7 to 9, had major response of 100%, 38%, 43%, and 40%. A category of patients at risk could not be identified concerning progression-free or overall survival, probably due to the low number of patients in each risk group.

### CRP response

**Figure 3:**
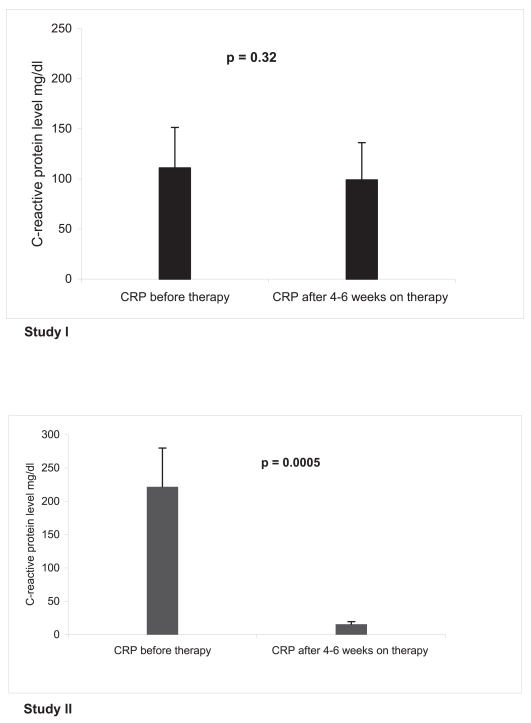
**C-reactive protein (CRP) response at study inclusion and after 4 to 6 weeks on treatment.** In study I mean CRP levels decline from 47.8 mg/dl (standard deviation 40.4 mg/dl) to 37.5 mg/dl (standard deviation 37.2 mg/dl), p = 0.33, in study II from 40.1 (standard deviation 38.0) to 4.9 (standard deviation 3.9 mg/dl), p = 0.0005. The mean initial CRP levels of cohort I and II were not significantly different, p=0.63.

CRP levels were available for follow-up in all patients assessable for response. Thirteen (72%) and 29 (93%) patients of cohort I/II had elevated CRP levels (CRP > 10 mg/dL) at study inclusion. The initial mean CRP levels of all assessable patients in cohort I/II (47.8 mg/dL/40.2 mg/dL) were not significantly different, p = 0.63. Exclusively in cohort II mean CRP levels declined significantly after 4–6 weeks on treatment (Figure 3): CRP levels decreased more than 30% in all patients with initially elevated CRP levels, in 24 of the 29 patients (83%) for more than 60%. The association of CRP decline and tumor response is shown Table 3. The CRP decline was in 22%/34% of the patients (protocol I/II) associated with an improvement of the ECOG status. Three patients in study II with assessed microbial infections grade 3 did not develop corresponding CRP elevations.

### Tolerability and safety

Both treatment regimens were aimed to facilitate long-term administration of the entire study medication by a scheduled early dose reduction in cases of toxicities > grade 1. Treatment-related toxicities are specified in Table 4a/b. In 4 (I) and 5 (II) patients (22%/16%), respectively, the therapy with the respective COX-2 inhibitor was discontinued during further treatment. Dose reductions of one or more drugs were performed in 72%/64% of the patients (study I/II), mainly due to capecitabine associated toxicity (Table 5a/b). Dose reduction of pioglitazone was not required.

Overall both therapy regimens were well tolerated. Due to renal insufficiency (4/4 patients) and hypertension (1/1 patient) the COX-2 inhibitors were discontinued after 3 to 5 treatment cycles. Cardiac adverse events were not observed. Two patients of study II discontinued therapy because of drug-related toxicities after 2.5 months (depression grade 3) and 6 months (hand-foot-syndrome grade 3). Hematotoxicity was generally mild.

Mild fever reactions and depression were specifically related to the additional administration of low-dose IFN-α. Transiently 6 and 7 patients, respectively (study I/II), received mild diuretic therapy (weight gain, edema). Fatigue developing after the initiation of treatment with rofecoxib/etoricoxib also was observed, albeit less frequently. All patients who experienced this side effect received the COX-2 inhibitor in the evening. Diarrhea, hand-foot-syndrome, nausea and vomiting were typically related to capecitabine.

## Discussion

The combination of the PPAR-γ agonist pioglitazone with a COX-2 inhibitor plus capecitabine resulted in negative response data and a moderate anti-inflammatory activity (study I). Study II assessed now, that the selected second transcription modulator, IFN-α besides pioglitazone, is complementary acting while tilting the systems biology of the tumor to a measurable response: Inflammation control in all patients with initially elevated CRP levels. Moreover, attenuation of the inflammatory activity was also established in single patients with clinically assessed infections. The successful disease modeling and the consecutively resulting tumor responses, as indicated by a steeply increasing response rate (0% vs. 48%, study I/II), are revealing a strong concerted biomodulatory activity of these at the respective doses in mono-therapy rather inefficacious drugs [Creagan et al. 1991].

Inflammation control seems to preceed tumor response and to occur independently of response behavior, either in cases with complete (pathologic) response or in those with rapidly progressive disease. Therefore, the CRP level represents no tumor marker, but an important biomarker for monitoring response to treatment of a cancer-related disease trait (tumor-related inflammation): A CRP decrease indicates the control of an either tumor-related or probably tumor-unrelated inflammatory process. The clinical equivalent is a corresponding improved ECOG status.

Approaches for a tailored modeling of tumor-associated disease traits, such as immunologic disorders and inflammation, owing to ubiquitous accessible transcription pathways in tumor and neighboring stroma cells (IL-2 and IFN-α receptor, retinoid X and glucocorticoid receptor) have been intensively studied in RCC. Previous phase III trials have tested the efficacy of various combinations of transcription modulators (IFN-α, IL-2, 13-cis-retinoic acid, glucocorticoids) plus/minus chemotherapy [Creagan et al. 1991; Atzpodien et al. 2004; no authors listed, 1999; Pyrhonen et al. 1999; Aass et al. 2005]. These studies are consistently demonstrating that just the concerted action of transcription modulators (mostly at high or maximal tolerable doses) is able to capture on a relatively small scale the strength of a negative regulation of growth promoting activities: The more transcription modulators are being included in a treatment arm (1 vs. >1), the better the respective response rates in comparison to the control arm, irrespective of the combinations or whether chemotherapy was added. Unfortunately, in all the same order the therapeutic index is steeply decreasing as indicated by an increasing incidence of grade 3 and 4 toxicities [Atzpodien et al. 2004]. Best response rates for high-dose IFN-α in these phase III trials are reported to range between 3–13% (PFS 2.7–5.0 months), for respective combination therapies between 8–26% (PFS 1.9–7.0 months). Most importantly, these phase III trials did not establish a similar stringent surrogate marker indicating a sufficient disease modeling either on an immunologic or inflammatory level. In the contrary, some trials are assessing inflammation as impeding factor for a successful therapy [Kedar et al. 2004].

Approaches targeting tumor-associated angiogenesis have recently been established in first-line therapy of metastatic RCC: Including multitargeted kinase inhibitors such as sorafenib or SU11248 in first and second-line therapy of RCC objective response rates are observed in up to 40% of the cases, and PFS is ranging between 6 to 8.3 months [Motzer et al. 2006; Ratain et al. 2006]. The specific inhibition of VEGF by the mono-clonal antibody bevacizumab induces response rates of 13% in patients with metastatic CCRC (PFS 8.5 months) [Bukowski RM et al. 2006]. Data of the current study are showing now, that anti-infammatory therapy in metastatic CCRC may be an additional important therapy approach besides the established angiostatic and immunologic therapy strategies.

The emerging pre-clinical data on the co-regulatory activities of transcription modulators underline our clinical observations as well as in vitro data on the reversal of IFN-α resistance following the addition of curcumin, a drug with PPARγ agonistic activity [Smith and O’Malley, 2004; Ogawa et al. 2005; Lee et al. 2005]. The extent of the combined activity of pioglitazone and IFN-α could be overestimated by possible chemosensitizing effects or other additive drug interactions with COX-2 inhibitors [Angelo et al. 2002; Rini et al. 2006; Kohno et al. 2005; Atzpodien et al. 2006]. Ongoing studies have to specify those effects. The multitude of activities ascribed for the different drugs used suggest that a precise mechanism for the schedules’ activity may be difficult to pin down [de Visser and Coussens, 2005]. However, the current study might indicate that (1) the capacity for a negative receptor-triggered regulation of pro-inflammatory responses is still preserved in the CCRC tissue (therapeutic plasticity) [Pfeffer et al. 1996; Tuna et al. 2004; Inoue et al. 2001]. (2) A possible starting-point for an effective therapeutic intervention has been recognized in the limitation of the activity of pro-inflammatory cytokines.

Targeted modeling of metastatic disease by focusing on the control of evolving pathophysiologically relevant disease traits during metastatic stage could be unifying therapeutic approaches for different tumor types. Combined transcription modulation is suggested to minimize regimen-related toxicity and to improve tumor response by disease modeling. This new therapeutic model might be easily integrated into more comprehensive treatment approaches.

## Figures and Tables

**Table 1:** Patient characteristics Study I and Study II.

	*STUDY I n = 18*		*STUDY II n = 33*	

Characteristics	No.	%	No.	%
Age, years				
Median	66		64	
Range	(53 – 85)		(48 – 78)	
Sex				
Male	13	72	21	64
Female	5	28	12	36
ECOG performance status				
0	3	17	7	21
1	12	67	20	61
2	3	17	6	18
Previous nephrectomy	16	89	30	91
Previous surgery of metastasis	3	17	17	52
Site of metastasis				
lung	9	50	27	82
liver	3	17	7	21
bone	4	22	12	36
adrenal	1	5	1	3
lymph nodes	4	22	12	36
contralateral kidney	3	17	5	15
ovary	1	5	-	-
pancreas	-	-	4	12
intestine	-	-	2	6
skin	-	-	2	6
thyreoid	-	-	1	3
Histologic grade				
0–3	14	77	26	79
4	1	6	3	9
unspecified	3	17	4	12
Motzer risk score				
low (0)	4	22	8	24
intermediate (1–2)	9	50	14	43
high (3–5)	5	28	11	33
Leibovich score				
Median	4.1		4.9	
Range	(− 1 to 9)		(− 1 to 9)	
Previous systemic treatment				
None	11	61	26	79
Interferon	3	17	1	3
Interferonα/Interleukin- 2	1	5	1	3
Velbe/IFNα/IL-2	2	11	3	9
5 Fluorouracil/IFNα/IL-2	1	5	2	6
Previous radiotherapy	3	17	6	18

**Table 2:** Response to treatment: Study I and Study II.

	*Study I n* = *18*		*Study II n* = *31*	

Response category	No.	%	No.	%
Complete	0	0	4	13
Partial	0	0	11	35
Stable	9	50	14	45
Progression	9	50	2	7

**Table 3:** C-reactive protein and tumor response.

	Number of patients (%)				
			with CRP response and		
	with elevated CRP levels	with CRP response	objective response	stable disease	progressive disease
***Study I***	13 (72)	9 (69)	0	9 (100)	0
***Study II***	29 (93)	29 (100)	13 (45)	14 (48)	2 (7)

CRP = C-reactive protein; CRP response: >30% decline of serum CRP level within 4–6 weeks on study medication.

**Table 4a:** Treatment-related toxicity Study I (n = 18).

	Toxicity Grade 1/2		Toxicity Grade 3		Toxicity Grade 4	
	
	No.	%	No.	%	No.	%
Diarrhea	4	(22)	-	-	-	-
Hand-foot-syndrome	2	(11)	1	(6)	-	-
Nausea/Vomiting	2	(11)	2	(11)	-	-
Edema	4	(22)	1	(6)	-	-
Hypertension	1	(6)	-	-	-	-
Creatinine elevation	7	(39)	-	-	-	-
Leukocyte counts	2	(11)	1	(6)	-	-
Anemia	5	(28)	-	-	-	-

**Table 4b:** Treatment-related toxicity Study II (n = 31).

	Toxicity Grade 1/2		Toxicity Grade 3		Toxicity Grade 4	
	
	No.	%	No.	%	No.	%
Diarrhea	6	(19)	-	-	-	-
Hand-foot-syndrome	8	(26)	3	(10)	-	-
Nausea/Vomiting	1	(3)	-	-	-	-
Edema	6	(19)	-	-	-	-
Creatinine elevation	8	(26)	-	-	-	-
Hypertension	1	(3)		-	-	-
Fever	2	(6)	-	-	-	-
Fatigue	3	(10)	-	-	-	-
Depression	-	-	1	(3)	-	-
Leukocyte counts	3	(10)	-	-	-	-
Platelet count	2	(6)	-	-	-	-
Hemoglobin level	3	(10)	-	-	-	-
Urinary tract infections	-	-	3	(10)	-	-

**Table 5a:** Dose modifications in Study I (n = 18).

Dose modification	No. of patients	
	Capecitabine	Etoricoxib/Rofecoxib
1g absolute twice daily	3	−/−
12.5 mg daily	-	−/7
Interruption of therapy (< 2 weeks)	3	2/7

**Table 5b:** Dose modifications in Study II (n = 31).

Dose modification	No. of patients		
	Capecitabine	interferon-α	Etoricoxib/Rofecoxib
1g absolute twice daily	12	-	−/−
3 MU three times a week	-	11	−/−
12.5 mg daily	-	-	−/9
interruption of therapy (< 2 weeks)	8	3	2/9
